# Relationship between health locus of control and sexual risk behaviour

**DOI:** 10.1186/1742-4690-9-S1-P62

**Published:** 2012-05-25

**Authors:** Enejoh Aromeh Victor, Karick Haruna

**Affiliations:** 1 Umsom - Institute of Human Virology, Nigeria, Fct Abuja, Nigeria; 2 Department of Psychology University of Jos Nigeria, Nigeria

## Background

Knowledge of the means of transmission and prevention of HIV/AIDS has been rated as the most important foundational factor for prevention. However studies have also shown that this knowledge does not always translate into reduced sexual risk behaviour (SRB). Perceived control over health status (“internal” locus of control) or attribution of health status to chance or fate (“external” health locus of control), HLC, is a psychological construct that has been shown to impact health outcomes. This study investigated the relationship between HLC and SRB. Hypothesis tested was, “individuals with an external locus of control will engage in more SRB compared with internals”.

## Methods

A cross sectional survey design was employed with 9 secondary schools in Jos,Plateau StateNigeria. Sample comprised of 361 students in senior secondary school. Health Locus of Control Scale measured attitude regarding perceived control over personal health, with individuals having either an internal or an external HLC while SRB was assessed using the Brief HIV Screener (BHS). Data was analyzed using the SPSS17.0. Descriptive statistics were computed while Analysis of variance was used to determine difference in SRB by HLC categories.

## Results

Respondents were 169 (46.8%) males and 192 (53.2%) females with a mean age of 16.9, age range 12-24 years. Mean HLC score was 38.54. When grouped into categories, 178 were internals while 183 were externals. Mean score on the BHS was 1.58, with a significant effect of gender. F (1,359) = 8.136, P < 0.01.Research hypothesis was supported; there was a statistically significant effect of HLC on SRB F (1,359) = 4.15, P < 0.05.

## Conclusion

Respondents who attributed their health status to chance or fate (external locus of control) significantly scored higher on SRB compared with internals. Prevention programs targeted at adolescents should also aim at internalizing their health locus of control.

